# Evaluation navigation controlled gate of aging spillway on cavitation damage

**DOI:** 10.1371/journal.pone.0311247

**Published:** 2024-10-09

**Authors:** Le Thi Thu Hien, Nguyen Van Chien, Nguyen Viet Duc

**Affiliations:** 1 Faculty of Water Resources Engineering, Thuyloi University, Ha Noi, Vietnam; 2 Hydraulic Construction Institute, Ha Noi, Vietnam; 3 Faculty of Civil Engineering, Thuyloi University, Ha Noi, Vietnam; CIFRI: Central Inland Fisheries Research Institute, INDIA

## Abstract

**Background:**

High-speed flow of clean water or water with sediment released from aging spillways may cause abrasion and cavitation on the concrete surface gradually. The occurrence of irregularities on the concrete surface can exacerbate the erosion problem. Which might jeopardize the safety of dams constantly, hence the rehabilitation efforts become urgent tasks in dam safety projects.

**Methods:**

This study employs a 3D Computational Fluid Dynamics (CFD) model to quantitatively analyze the cavitation risk on the aging concrete surface of the Chay 5 spillway in Ha Giang, Vietnam, under various operation scenarios. There are two standards used to measure cavitation: the cavitation index (*σ*) which indicates the danger due to the drop of pressure in rapid flow, and the new gasification index (*β*) which takes into consideration the formation and collapse of bubbles behind asperities.

**Results:**

Three extreme flood cases may not result in potential cavitation because both *σ* and *β* exceed critical thresholds. Regarding the six controlled gate scenarios with normal water level, the *σ* profiles are approximated 1,0 showing a low likelihood of cavitation damage while the *β* values are smaller than 0.8, indicating a considerable risk of cavitation. Besides, the opening height of 100 cm poses the greatest risk of creating severe cavitation erosion in the concave area and slope portion. The flip bucket experienced the most vulnerable cavitation when the opening height is 400 cm. In addition, an approach to spillway surface rehabilitation involving specialized mortars has been presented.

**Conclusion:**

For aging conveyance structure, gasification index (*β*) takes into account irregularities surface, providing a more comprehensive assessment of the likelihood of cavitation damage than cavitation index (*σ*). After rehabilitation with anti-shrinkage high abrasion resistance mortar, the entire spillway surface is smooth. This allows for reducing the cavitation risk and improvement of life service thereof.

## 1. Introduction

Various manifestations of aging signs in concrete structures can be listed: cracking, expansion, spalling, cavitation and abrasion, etc. The general conclusion drawn is that nothing can be made to extend the lives of old dam indefinitely, but a lot can be done to elongate their service life with repair and upgrading works until technical considerations prove them unfeasible or their cost become prohibitive [1]. In several reasons causing concrete surface failure in structures conveying high-velocity flows, such as: deterioration of construction materials, design flaws, lack of regular maintenance, cavitation and abrasion can indeed be main reasons [1–9]. Abrasion is the mechanical wearing down of a surface due to friction and the impact of solid particles carried by the high speed flow. This can result in a roughened surface, loss of material, and reduced structural integrity over time [1, 10]. In contrast, cavitation occurs when water flows at very high speeds, it can create vapor bubbles that collapse and generate powerful shock waves, causing surface pitting and erosion [4, 10]. Abrasion phenomena is often experimentally detected or visualized for long period while cavitation issue can be numerically predicted. Potential cavitation risk has been assessed by some indicators. Cavitation index (*σ*) was used to assess cavitation risk due to the drop of pressure in rapid water flow and it has been used prevalently by numerical models [2, 4, 10, 11]. On the other hand, the development of cavitation due to the effect of different types of irregularity was firstly described in [12] by using the concept of gasification index (*β*). Nguyen Chien (2016) used this parameter to estimate cavitation damage on a high-head dam [13]. Nevertheless, the hydraulic properties derived from the 1D numerical model were based on depth-averaged values. Consequently, it was unable to calculate hydraulic parameters in close proximity to the bottom surface, resulting in a less accurate prediction of the danger of cavitation. The measurement of gasification index has been rare since it requires prolonged measurement of surface roughness, as well as correct estimation of hydraulic parameters near the rough areas.

In fact, utilizing 3D CFD models can be a cost-effective way to investigate potential cavitation risk on conveyance structures before implementing physical modifications [10–21]. Among several 3D CFD models, advanced capabilities of Flow 3D in multiphase flow, sediment transport modeling, and fluid-structure interaction make it a highly suitable tool for variety of applications, such as dam break analysis, flood modeling, and environmental hydraulics, etc. Besides, the precision, flexibility, and user-friendly features of Flow 3D enable researchers and engineers to achieve accurate and reliable results, leading to better-informed decisions and optimized designs [4, 13, 18, 22–28]. Several researchers used cavitation index (*σ*) to assess cavitation phenomena by applying 3D CFD model [2, 4, 22]. The use of this index suggests that the assessments were focused on predicting potential cavitation without explicitly considering the presence of irregularities in aging concrete surfaces. Prior studies commonly examine the potential cavitation risk in situations of extreme floods. The comprehensive consideration of scenarios using navigation controlled gates may be inadequate. Therefore, beside using cavitation index (*σ*) we examine the gasification index (*β*) as a means of measuring the danger of cavitation caused by irregularities on aging surfaces in different operation scenarios.

Run-of-the river projects required very limited storage to generate low-cost clean energy, which have often released water and sediment together [29]. During early flood events, controlled releases of water, along with sediment, are initiated to mitigate the buildup of sediment in the reservoir. After long service of old dams, where roughened concrete surfaces have been developed already, the impact of abrasion and cavitation erosion damage will be intensified [10]. In Vietnam, this kind of project like cascade hydropower plants have prevalently constructed in mountainous rivers such as: Nam Chien, Chieng Muon, Nam Chien II in Chien river (Son La province); Chay 3, Chay 5, Chay 6 in Chay river (Ha Giang province). The hydropower plant Chay 5 is the run-of-river project, which has been built since 2011 [30]. Recently, scuffing, trenches, and erosion can indeed pose serious risks to the safety and functionality of spillways and associated structures. Therefore, addressing these issues promptly is crucial to prevent further damage and ensure the long-term integrity of this work.

Some novelty achievements in this study can be listed:

- Analysis of cavitation mechanism on aging spillway surface due to release rapid water flow with sediment.
- The cavitation index (*σ*) and gasification index (*β*) are important for measuring cavitation risk, especially in three extreme flood scenarios like the Chay 5 spillway. Although these indicators show that cavitation may not be the main cause of spillway surface erosion under such conditions.
- Accounting for six navigation controlled gate scenarios with normal water levels, the *β* index is a more precise measure of cavitation risk in specific navigation scenarios than the *σ* index because it takes into account irregularities in the surface. These indicators also identify the specific places on the spillway surface that are susceptible to cavitation. Furthermore, they indicate the operation situation that yields the greatest likelihood of cavitation risk in each specific region. Hence, it is imperative to give priority to this aspect during the process of repairing, maintaining, and developing navigation strategies.
- Specialized mortars and repairing technique are introduced to rehabilitate the current rough surface of the damaged spillway. The outcome would be the smooth surface, which prevents from the disintegration due to the turbulence and cavitation caused by the fast-flowing liquids along the surfaces [30, 31]. The spillways after rehabilitation with a protective layer made of the mortars could sustain from the further detrimental effect, consequently enhance life service thereof.

## 2. Methodology and case study

### 2.1. Methodology

The Navier-Stokes equations are a set of partial differential equations that describe the motion of liquids and gases [19]. The Navier-Stokes equations play a crucial role in understanding fluid dynamics, turbulence, and the behavior of fluids in a variety of engineering and environmental contexts [14]. The general form of these equations for an incompressible fluid in three dimensions is given by:

∇.v=0 (1)

$$\rho \left(\frac{\partial v}{\partial t}+v.\nabla v\right)=-\nabla p+\rho g+\mu \nabla^{2}v$$ (2)

The Eq (1) represents the conservation of mass and states that the divergence of the velocity field while the Eq (2) describes the conservation of momentum.

The Volume of Fluid (VOF) method is a numerical technique used to simulate the interface between two immiscible fluids, such as air and water, in computational fluid dynamics. The method is particularly useful for studying fluid flows where the location and shape of the fluid interfaces are important, such as in multiphase flows and free-surface flows. Besides, turbulence is a complex and chaotic phenomenon that involves the irregular motion of fluid particles, leading to fluctuations in velocity and pressure. There are different approaches to model turbulence in CFD simulations, and these can be broadly categorized into two main types: Reynolds-Averaged Navier-Stokes (RANS) models and large eddy simulation (LES) models. The Renormalization group (RNG), which is of the RANS type, is selected due to its capacity to simulate flow over hydraulic works.

The integration of VOF particles with sharp-interface tracking VOF methods in Flow 3D represents a significant advancement in CFD. In addition, Flow 3D allows for flexible and realistic boundary conditions, which can be tailored to specific project requirements. This ensures that the model accurately represents the physical conditions of the spillway and surrounding environment. Besides, Flow 3D supports adaptive mesh refinement, allowing for high resolution in areas of interest while keeping computational costs manageable. This ensures detailed and precise simulations of critical regions.

However, like many CFD tools, Flow 3D may require significant computational resources like memory and processing power for high-fidelity simulations or simulations of large-scale hydraulic systems [32].

### 2.2. Case study

#### 2.2.1. Location and configuration of Chay 5 spillway

**Fig 1:**
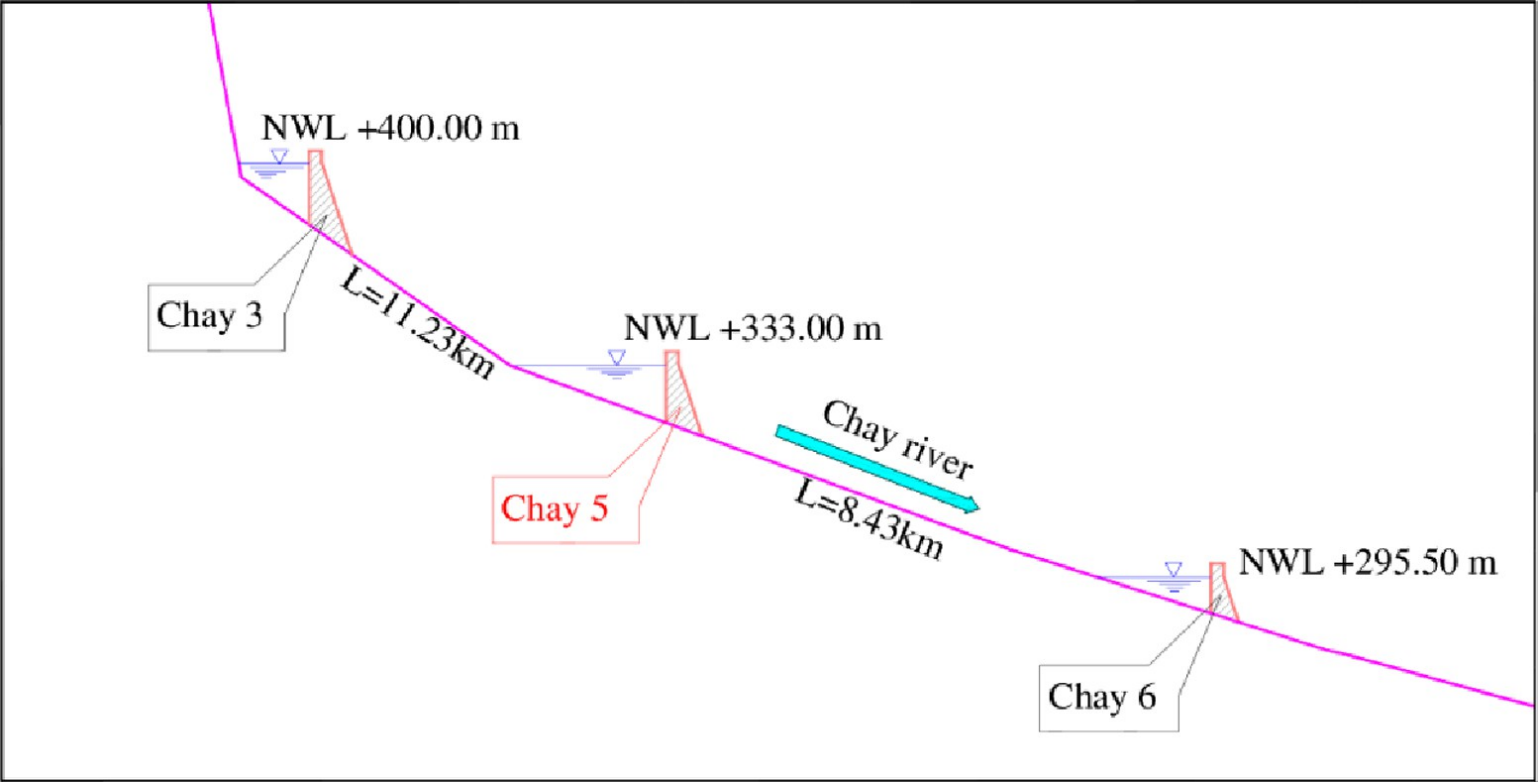
Schematic of cascade hydropower stations in Chay river.

The suitability of the Chay River’s steep terrain for small cascade hydropower stations, namely Chay 3, Chay 5 and Chay 6 is advantageous (Fig 1). The release of water mixed with sediment through spillways during early flood events is a common practice to prevent sedimentation in the reservoirs. This approach helps maintain reservoir capacity and prevents the buildup of sediment, which can impact both water storage and power generation efficiency.

**Fig 2:**
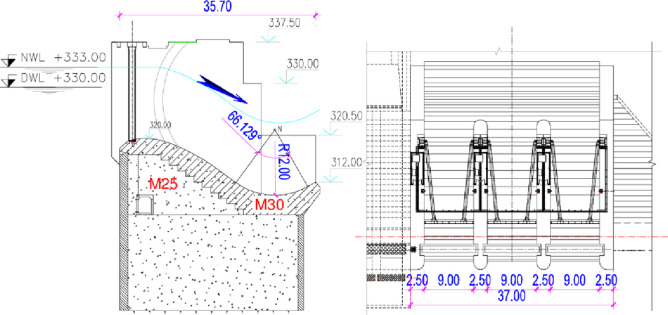
The configuration and components of Chay 5 spillway (dimension in meters).

The total volume storage of Chay 5 reservoir is 4.86×10^6^ m^3^ and its effective volume is 1.28×10^6^ m^3^. Its spillway has some parameters, including: dead water level (DWL): 330 m; normal water level (NWL): 333 m; inflow design flood (P = 1.0%) (IDF): + 334.13 m; extreme water level (P = 0.2%) (EF): + 335.31 m and the crest elevation: 320 m. Dimensions of ogee spillway: three control gates (*B×H*) = 9.0 m×13.5 m (Fig 2).

#### 2.2.2. Realistic condition of the concrete surface of Chay 5 spillway

**Fig 3:**
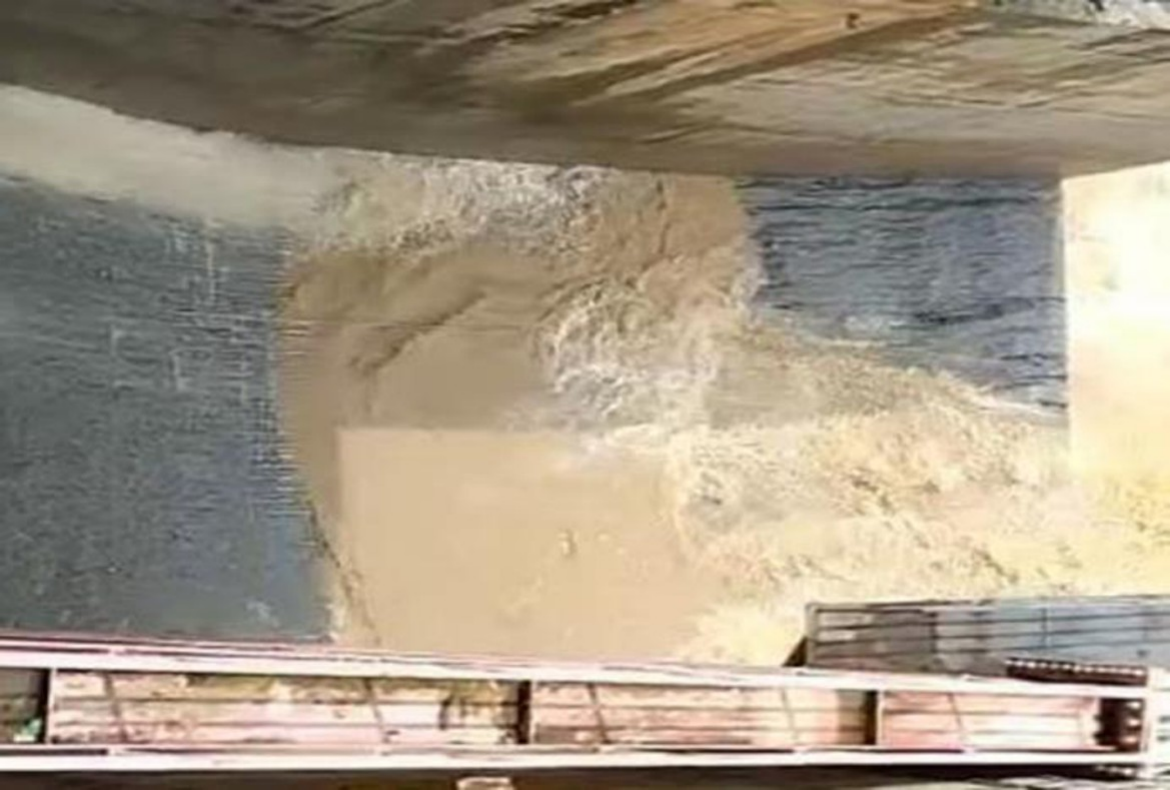
Chay 5 spillway releases water with sediment.

According to [33], before high-head conveyance structures started working, the maximum roughness height on the concrete surface should be smaller than 4.0 mm and all local asperities must be removed to mitigate abrasion or cavitation phenomena. However, after 10 years of operation there are concerns about the condition of the spillway surface of Chay 5, with indications of strong deterioration and dense erosion holes at slope, concave segments and flip bucket (Fig 3). Particularly, concrete is removal and anchored bars and wire mesh are exposed at slope area. This deterioration may impact the performance and overall safety of structurer. Moreover, there is a recognition of a recurring issue related to erosion on spillway surfaces in cascade works. Therefore, understanding the reasons for induced erosion on the spillway surface is essential to propose effective solutions for the rehabilitation of the Chay 5 spillway. The insights gained from this analysis can also be valuable for other similar projects, ensuring broader applicability and improved infrastructure resilience.

### 2.3. Model set up

**Fig 4:**
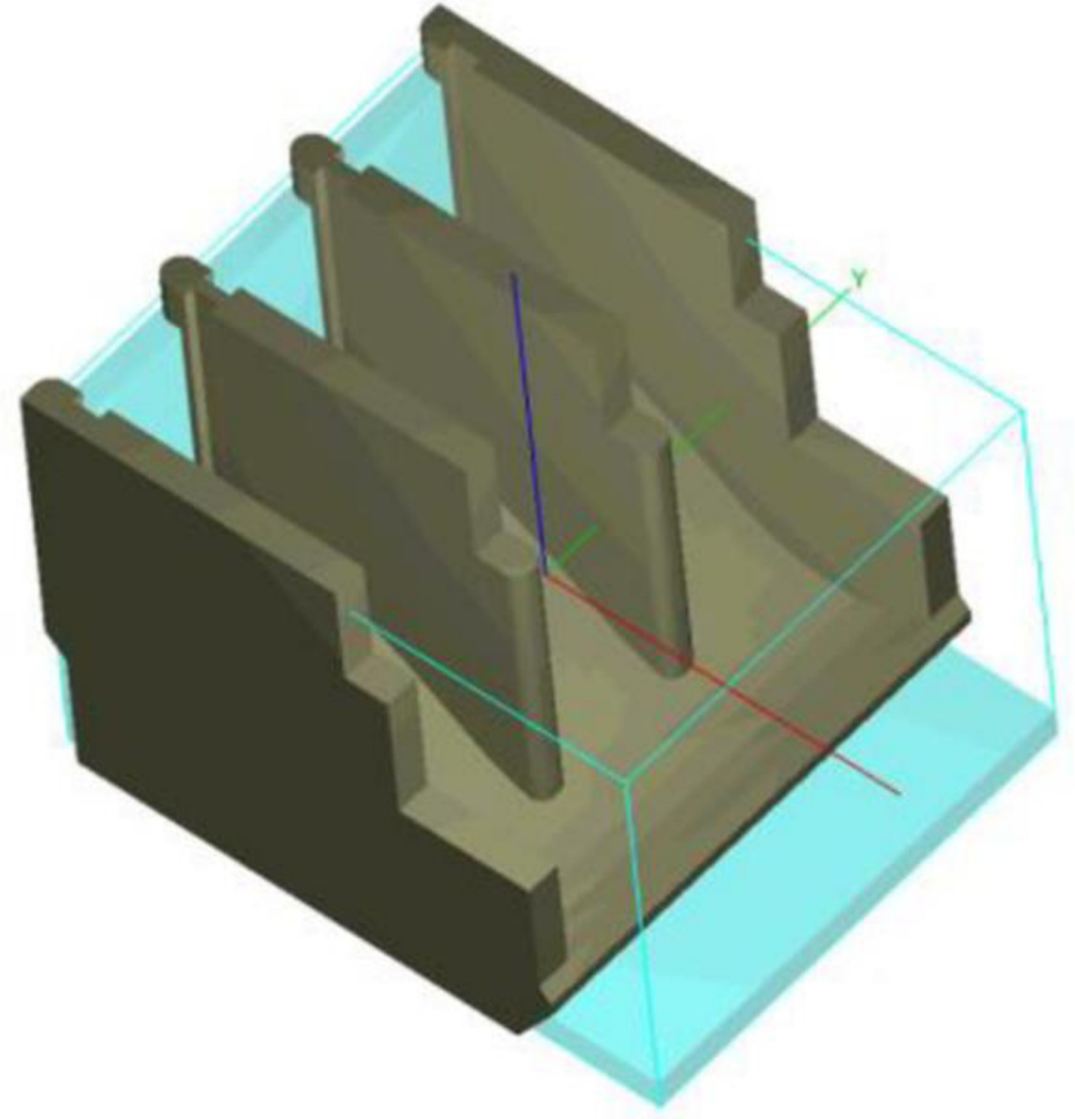
Computational domain of Chay 5 spillway.

The domain, including all vents of the spillway and extends downstream is discretized by structured mesh. The upper boundary is specified as a specific pressure, and there are alternative constant water levels taken from Table 1. These data are also initial water levels at upstream and downstream of spillway. The lower boundary is flow-out. Two side boundaries are wall (Fig 4). The roughness coefficient is taken by 0.017. The computational domain is discretized by structural mesh and mesh convergence is analyzed and validated in detail at two subsections 3.1.1 and 3.1.2.

**Table 1:** Operation cases of Chay 5 spillway in numerical simulation.

N^0^	Operation cases	Upstream water level (m)	Downstream water level (m)	Opening height (cm)
1	NWL	+ 333.00	+ 306.35	Fully open all gates
2	IDF	+ 334.13	+ 308.13	Fully open all gates
3	EF	+ 335.31	+ 311.82	Fully open all gates
4	NWL	+ 333.00	+ 306.35	25
5	NWL	+ 333.00	+ 306.35	50
6	NWL	+ 333.00	+ 306.35	100
7	NWL	+ 333.00	+ 306.35	150
8	NWL	+ 333.00	+ 306.35	200
9	NWL	+ 333.00	+ 306.35	400

### 2.4. Theoretical mechanism and cavitation standards

#### 2.4.1. Theoretical mechanism

For aging concrete conveyance structure, lack of regular maintenance, such as failure to remove debris or repair damaged sections promptly, can lead to accelerated erosion [10]. The gradual deterioration of the concrete surface over time can be attributed to abrasion and cavitation caused by the release of high-speed water flow with sediment.

**Fig 5:**
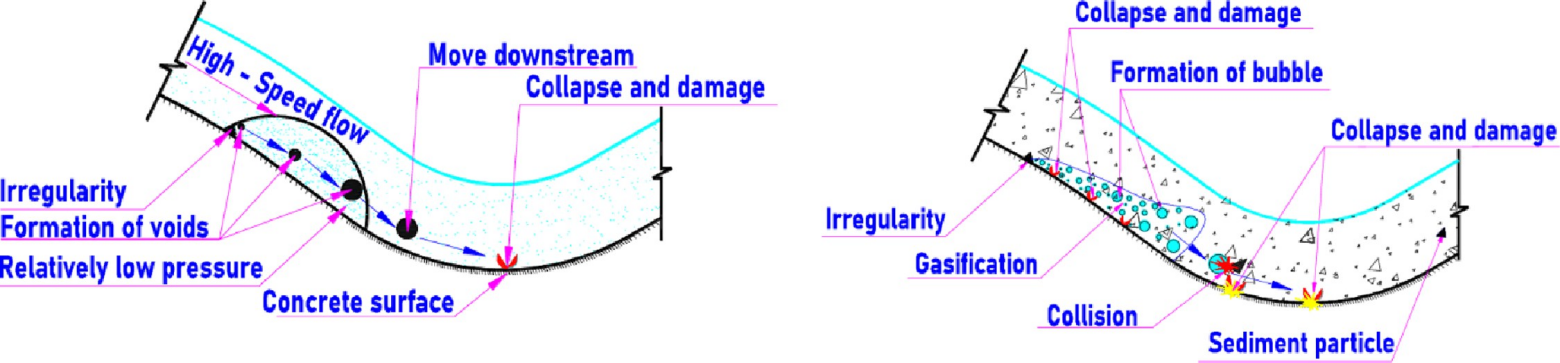
Cavitation formation and damage mechanism for a spillway surface. a) Bubbles broken due to drop of pressure [11]. b) Bubbles broken due to collide with sediment particles.

In the clean water, the formation and damage mechanism of the cavitation induced by irregularities in a spillway surface was explained Fig 5A [11]. A relatively low-pressure zone occurs behind the irregularities when the high-speed flow passes an irregularity on the solid surface. Vapor bubbles nucleate in these low-pressure zones. This nucleation is facilitated by impurities or microbubbles already present in the water. These bubbles grow as the pressure remains below the vapor pressure, often expanding until they encounter regions of higher pressure. Consequently, they collapse when subjected to the higher pressure in the flip bucket [11, 33]. If the change of pressure is not sufficient, bubbles may be not broken, hence cavitation may be not occurred [34]. In the high-speed flow of water mixed sediment, however, these bubbles may be collapsed due to colliding with sediment particles (Fig 5B). The particle-bubble collision process was described in several researches, [35, 36]. The collapse generates intense shock waves and micro-jets, which impact the spillway surface. These impacts create extremely high local pressures that can exceed the material strength of the spillway surface [31, 37]. Over time, the formation of pits and cracks increases the roughness of the spillway surface. This enhances local turbulence, creating more favorable conditions for cavitation, thus perpetuating the damage cycle. Besides, rougher surfaces experience more intense cavitation, leading to accelerated erosion and material loss.

#### 2.4.2. Cavitation standards

*a*. *Cavitation index (σ)*. The cavitation index (*σ*) is typically used to investigate cavitation risk on design hydraulic structures without specifically considering the effects of irregularities [4]. It is expressed by Eq (3):

$$\sigma =\frac{p_{o}-p_{v}}{\rho V^{2}/2g}$$ (3)

According to [38], if *σ* < 1.0, the local pressure approaches or drops below the vapor pressure, making the formation of vapor bubbles more likely, indicating a high risk of cavitation. Smaller value of *σ* leads to greater potential for cavitation damage [38].

*b*. *Gasification index (β)*. On the other hand, the gasification index (*β*) becomes more crucial in situations the impact of roughness in aging conveyance structures is inevitable. The stages of gasification can be divided into two stages:

- Inertial cavitation can also occur in the presence of an acoustic field. Microscopic gas bubbles that are generally present in a liquid will be forced to oscillate due to an applied acoustic field. If the acoustic intensity is sufficiently high, the bubbles will first grow in size and then rapidly collapse.
- Ultrasonic cavitation inception will occur when the acceleration of the ultrasound source is enough to produce the needed pressure drop. This pressure drop depends on the value of the acceleration and the size of the affected volume by the pressure wave.

Wijngaarden (2016) showed that, fluid motion is caused in the rigid boundary by a component that is perpendicular to the wall. This component is known as the impulse of the bubble and represents the inertia of the fluid around the bubble as it moves towards the wall. In this case, the micro jets are likewise oriented away from the wall, and shock waves prove to be more efficient for the reason of cleaning [39, 40].

Besides, according to [12, 36], the condition to generate bubbles at irregularities surface in the rapid flow is:

$$\beta =\frac{K}{K_{c}}\;\;\;<1.0$$ (4)

If *β* is in the range of 0.8 to 1.0, the acoustic stage is produced; from 0.1 to 0.8, the acoustic stage is developed while *β* < 0.1, the ultrasonic cavitation inception occurs. where: *K* is the coefficient of bubble generation:

$$K=\frac{h-h_{v}}{V_{b}^{2}/2g}$$ (5)

$$\text{with}\;V_{b}=\frac{V}{\varphi_{v}}\sqrt{\xi_{1}.\xi_{2}}$$ (6)

*ξ*_*1*_ and *ξ*_*2*_ are parameters depending on the ratios (*Z*_*m*_*+Δ*)*/Δ* and *δ/Δ*, respectively, which are taken from [41].

In case of free surface flow and rectangle cross section, *ϕ*_*v*_ can be estimated by formula (7):

$$\varphi_{v}=\frac{1}{Bh}\left\{\left(h-\delta \right)\left(B-2\delta \right)+\frac{\delta^{2}}{\text{ln}\frac{\delta }{\Delta }+3}\left[\frac{B+2h}{B}\left(\text{ln}\frac{\delta }{\Delta }+2\right)-2\text{ln}\frac{\delta }{\Delta }-5\right]\right\}$$ (7)

The critical value *K*_*c*_ depends on the shape of singular roughness or uniformly distributed ones like: offset-into the flow, offset-away from the flow, voids or grooves and protruding joints, etc.

*K*_*c*_ can be estimated by the formula (8) [12, 36]:

$$K_{c}=2.24{\left(\frac{L_{m}}{Z_{m}}\right)}^{-0.5}$$ (8)

The approach includes four steps:

- According to the particular operating procedure of specific hydraulic structurer, the operation scenarios are selected. Besides, a 3D CFD software package is also selected to simulate hydraulic characteristics.
- Based on realistic situation of concrete surface, the properties of roughness, irregularities are collected.
- Cavitation index (*σ*) and gasification index (*β*) are suggested to analysis the cavitation risk associated with different operation scenarios. Hence, the cavitation potential zone is indicated. The probability of cavitation risk corresponding with each operation scenarios is quantified.
- Proposal of the rehabilitation technique based on the type and level of damage.

**Fig 6:**
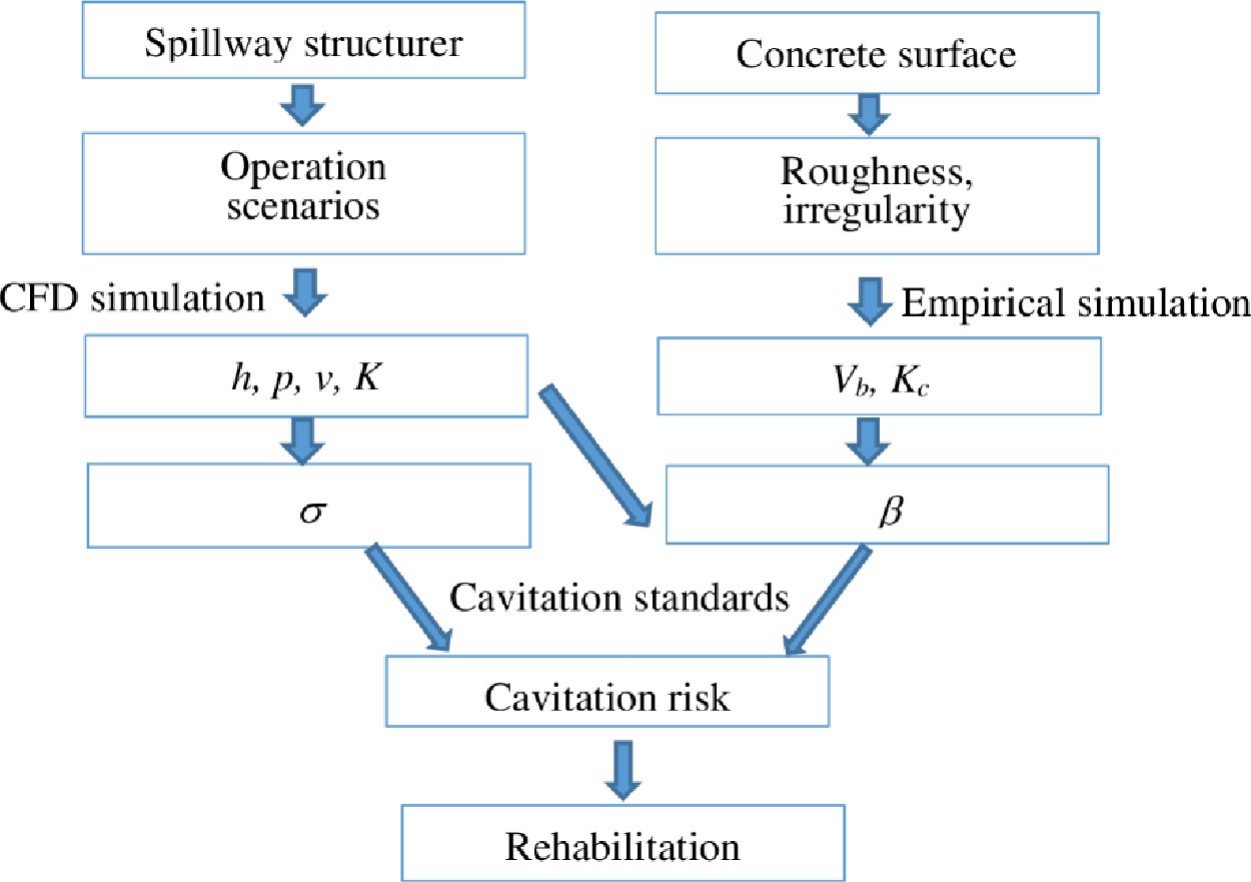
Flowchart for cavitation risk analysis of aging spillway.

Fig 6 describes the general procedure for cavitation analysis based on the CFD technique.

## 3. Results and discussion

### 3.1. Mesh convergence analysis and validation

#### 3.1.1. Mesh convergence analysis

The Grid Convergence Index (GCI) is a metric used in CFD studies to assess the convergence and quality of numerical solutions obtained using different grid sizes. It helps in estimating the error introduced by the discretization of the computational domain. So, a small GCI value means the solution is in the asymptotic range. Three different structured meshes of 0.2 m; 0.3 m and 0.45 m corresponding with fine, medium, and coarse meshes, respectively, are employed. Similarly to [42], in this study, the numerical discharge released from entire spillway is used to compute GCI value (Table 2).

**Table 2:** Mesh convergence analysis.

N^0^	Cell size (m)	Discharge (m^3^/s)	GCI (%)
1	0.20	2525.1	
2	0.30	2531.6	0.173
3	0.45	2550.2	0.493
GCI_32_/r^p^GCI_21_			0.997

As can be seen from Table 2, the value of GCI reduces from 0.493% to 0.173% for GCI_32_ and GCI_21_, respectively. It can be said that the grid-independent solution is nearly achieved and does not need to carry out further mesh refinement. Moreover, the value of GCI_32_/r^p^GCI_21_ close to 1.0 indicates that numerical solutions are within the asymptotic range of convergence. As a result, the fine grid spacing of 0.2 m is sufficient to obtain a reliable numerical solution in the present study.

#### 3.1.2. Validation

According to [4], the total discharge released from unsubmerged ogee spillway is expressed by the Eq (9):

$$Q_{theory}=\varepsilon m\sum b\sqrt{2g}H_{0}^{3/2}$$ (9)

where: $H_{0}=H+\frac{V_{0}^{2}}{2g};\;\;\;\sum b=3\times 9=27\left(m\right)$

*ε* and *m* are taken from TCVN 9147–2012 [43].

To discorver the suitable cell size of 0.2 m in modelling the flow over Chay 5 spillway, the accuracy of modeling results should be investigated. By comparing numerical results of total discharge released by fully open all gates of spillway under three extreme water levels are compared with theoretical solutions. Relative error *E* (%) is estimated by Eq (10):

$$E=\frac{\left|Q_{\mathrm{mod}el}-Q_{theory}\right|}{Q_{theory}}.100\;\left(\%\right)$$ (10)

Certainly, the good matching of numerical results with theoretical ones is observed because all relative error (*E*) values are smaller or approximately equal to 1.0% (Table 3). This point indicates that for simulating hydraulic characteristics across the entire domain, this cell size is deemed suitable. However, for a more accurate performance of hydraulic characteristics within a one-meter width of the spillway, a finer refinement mesh of 0.04 m is selected to simulate.

**Table 3:** Comparison numerical and theoretical discharge.

N^0^	Cases	Upstream water level (m)	*H* (m)	*P* (m)	*Q*_*theory*_ (m^3^/s)	*Q*_*model*_ (m^3^/s)	*E*(%)
1	NWL	+ 333.00	13.00	33.5	2513.49	2518.0	0.16
2	IDF	+ 334.13	14.13	33.5	2871.54	2880.0	0.29
3	EF	+ 335.31	15.31	33.5	3267.76	3303.0	1.08

### 3.2. Potential cavitation analysis of operation scenarios

#### 3.2.1. Hydraulic characteristics

High-speed flow can lead to more significant abrasion, exposing the underlying substrate surface over time. In high-head spillways, the magnitude of velocity varying between 12 m/s to 15 m/s is within the cavitation-prone range, and serious damage may occur if the velocity exceeds 22 m/s [1]. Therefore, investigation velocity profile along spillway surface is the first step to predict the potential erosion risk.

**Fig 7:**
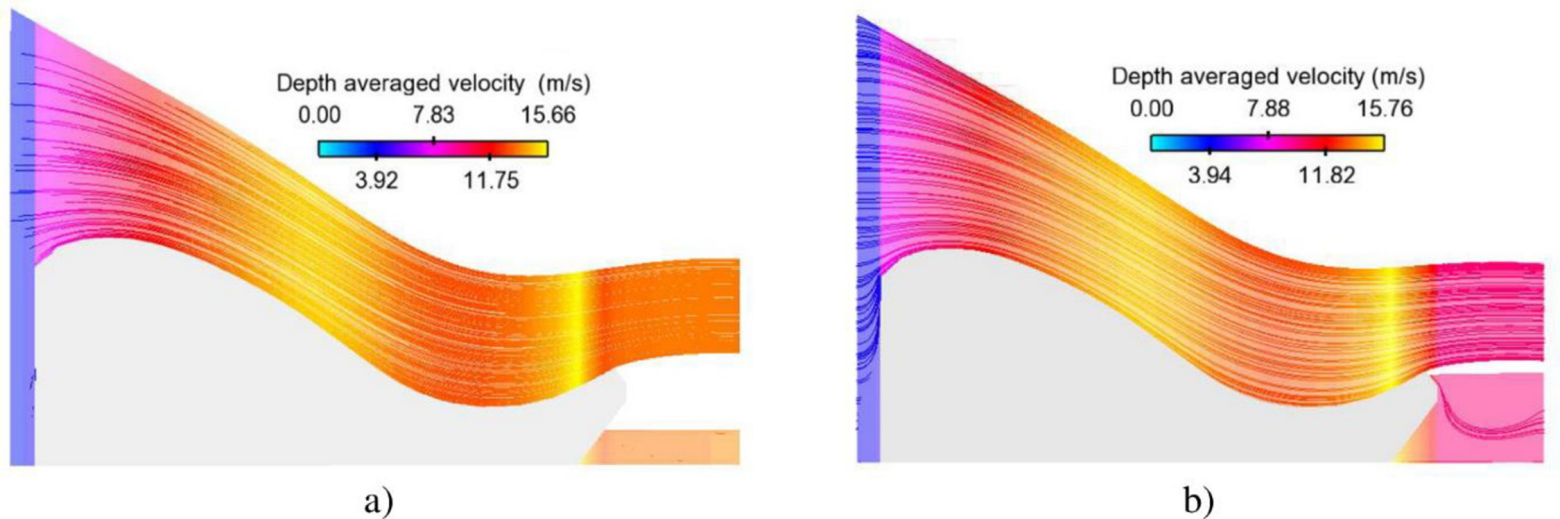
Velocity distributions along Chay 5 spillway surface. a) IDF case; b) EF case.

**Fig 8:**
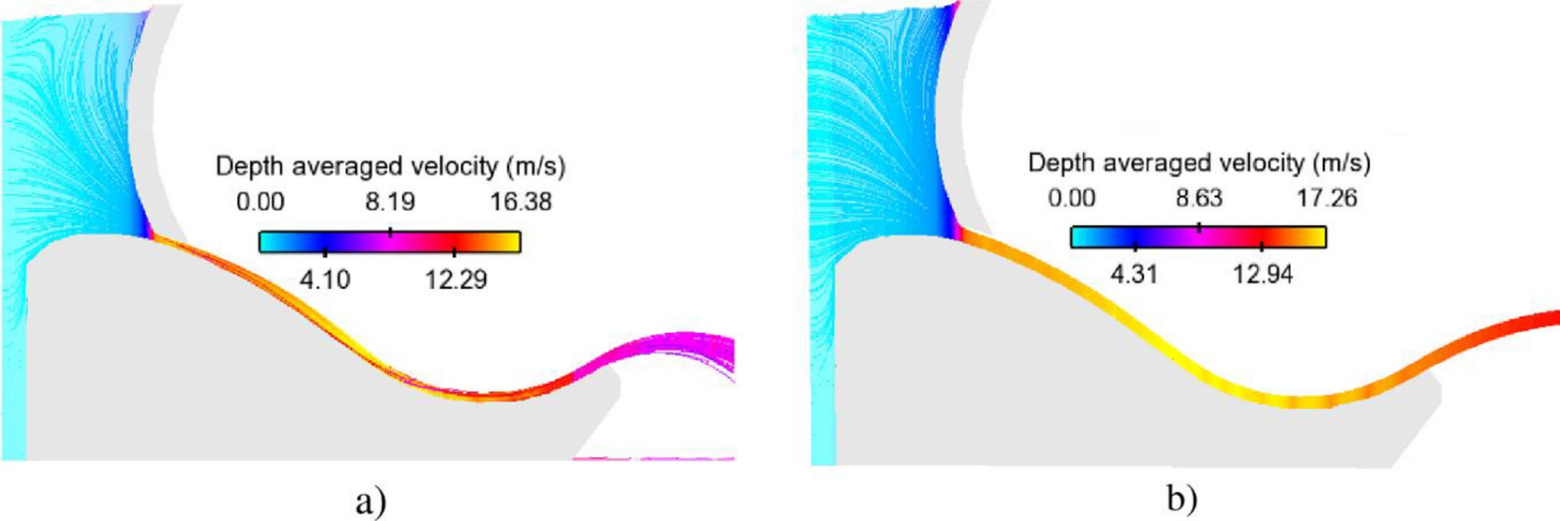
Velocity distributions along Chay 5 spillway surface. a) Opening height of 50 cm. b) Opening height of 100 cm.

Fig 7 shows the depth-averaged velocity profiles resulting from two extreme floods IDF and EF. Maximum values occurring at slope segment and flip bucket are 15.66 m/s and 16.14 m/s, respectively, which may lead to damage on the concrete surface. Despite the initial water level for group of controlled gate scenarios being smaller than the aforementioned cases, the magnitude of velocity in the former group is greater than that of the latter (Fig 8). Which may have implications for the structural integrity or safety of the system under consideration.

Therefore, the probability of cavitation damage occurring in the controlled gate cases may be greater than those in extreme floods. The following part analyses carefully the potential cavitation damage by using cavitation standards presented in subsection 2.4.2.

#### 3.2.2. Assessment cavitation damage level under alternative navigation scenarios

**Fig 9:**
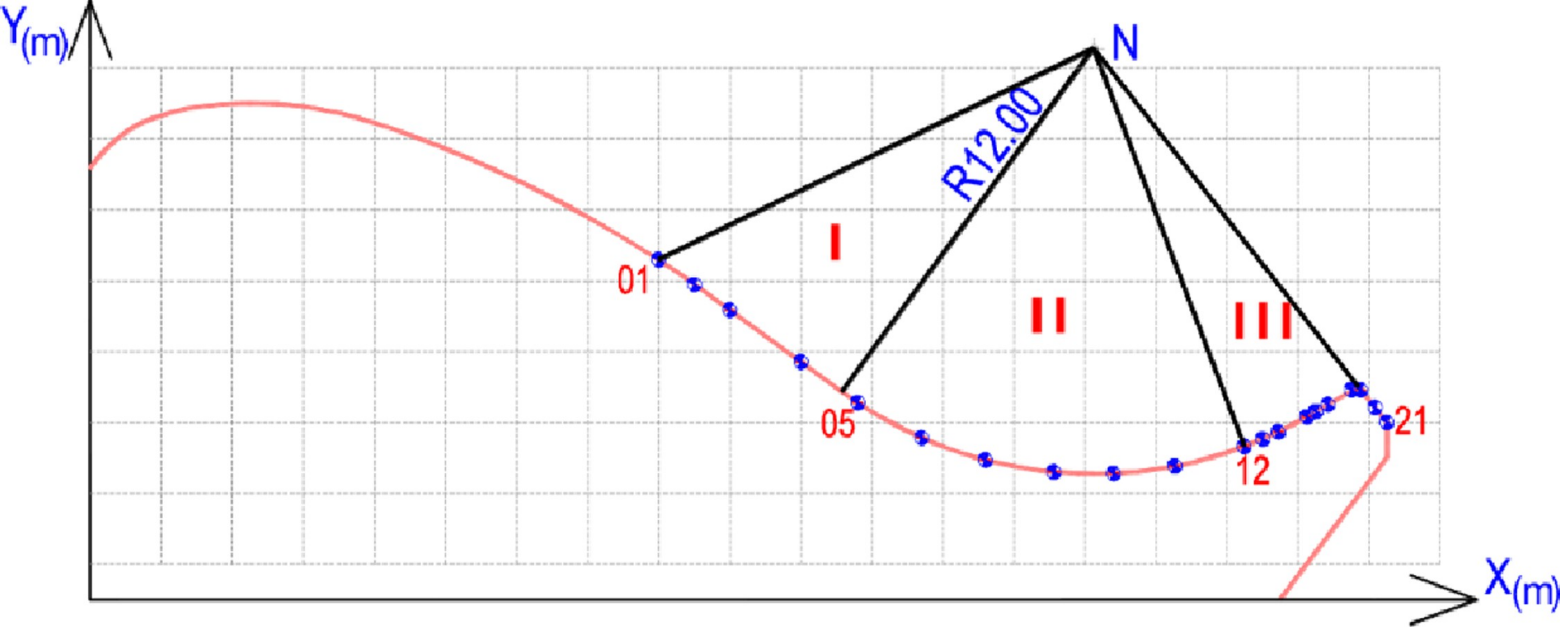
Location of studied sections.

The information suggests that there is significant damage in the 20.6 m length of the Chay 5 spillway. This damage has developed over the years, resulting in the removal of concrete surface and the formation of roughness and irregularities, as depicted in Fig 9. Additionally, reinforced steel is partially exposed at the slope section and completely exposed on the concave segment and flip bucket (Fig 3). Despite these issues, Chay 5 spillway has been working without any apparent warning or restriction. Cavitation and abrasion are potential risks that can arise from such structural damage, and it is important to identify the most dangerous navigation case to address these issues effectively.

Based on the realistic damage situation of spillway surface, specific area is divided into three zones: zone I at slope segment; zone II at the concave segment and zone III at the flip bucket (Fig 9). Some parameters of roughness and irregularities of these zones are indicated in the Table 4.

**Table 4:** Parameters of roughness and asperities at damage areas.

Sections	*x/L*	Zone	*Δ* (mm)	*L*_*m*_ */Z*_*m*_	*K*_*c*_
1–4	0.45–0.55	I	6	0.873	2.4
5–12	0.55–0.90	II	6	0.596	2.9
13–21	0.90–1.0	III	6	0.409	3.5

**Fig 10:**
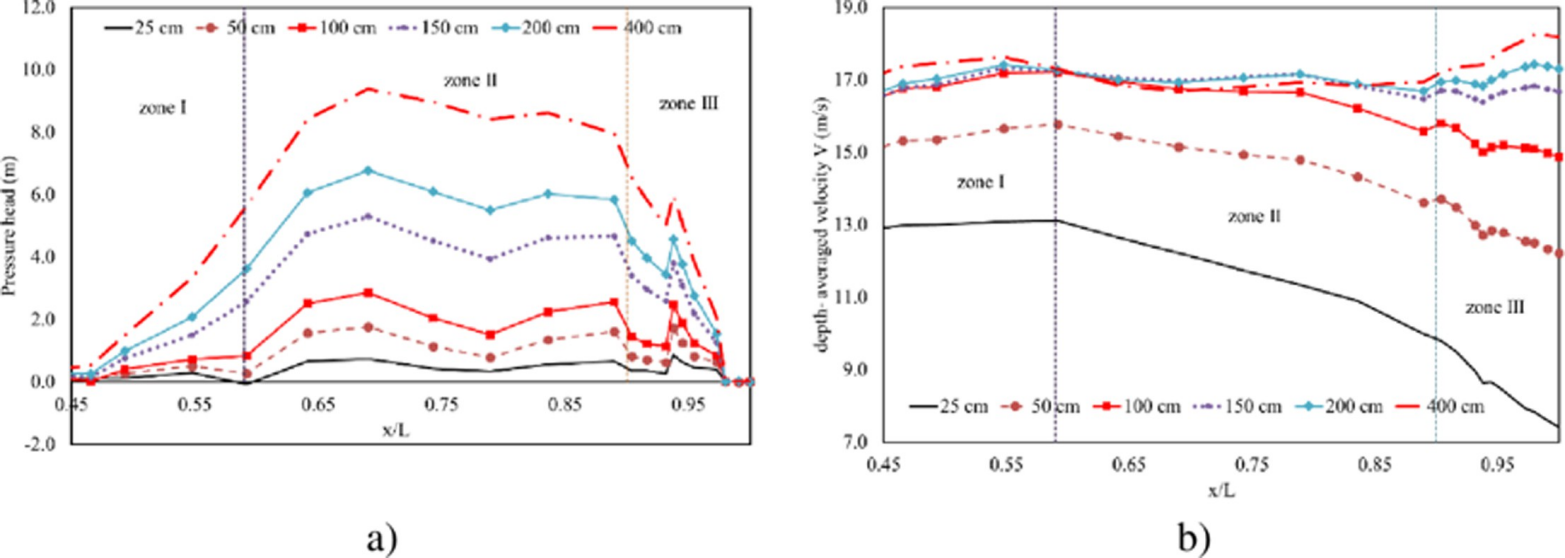
Hydraulic characteristics at three zones. a) Pressure head profiles. b) Depth-averaged velocity profiles.

Studying the velocity distribution across the spillway can reveal areas where high velocities might induce cavitation. Understanding how pressure changes with varying gate heights can identify zones susceptible to cavitation damage. Within 6 navigation cases of controlled gate, the higher pressure head observed at zone II with greater gate opening heights is likely due to increased water flow and velocity passing through this section. But, this value is almost equal zero at the nose of flip bucket (Fig 10). Two distinct groups, Group A and Group B, are identified by opening heights. Group A includes smaller opening heights (25 cm, 50 cm, and 100 cm), while Group B comprises larger opening heights (150 cm, 200 cm, and 400 cm). For Group A, there is a slight increase in water depth and a rapid decrease in velocity across all zones. Noteworthy differences in velocity values are observed among the three openings, suggesting that opening height plays a significant role in influencing flow characteristics. In contrast, Group B exhibits a notable difference in flow depth among all scenarios, indicating that opening height is a crucial factor influencing water depth. Despite variations in opening heights, the depth-averaged velocity remains consistent in both zones I and II. However, in zone III, there is an observable increase in velocity with greater opening heights. These findings emphasize the importance of considering the impact of gate opening heights on water depth and velocity profiles.

**Fig 11:**
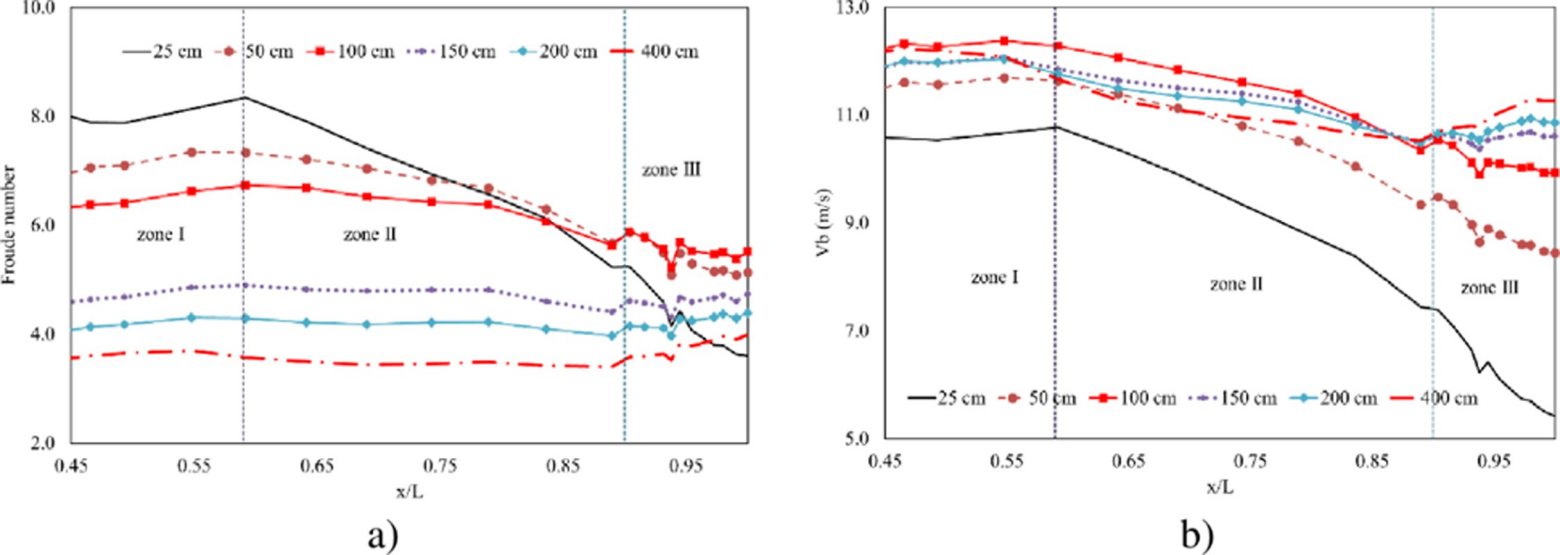
Hydraulic characteristics at three zones (continued). a) Froude number profiles. b) Boundary velocity profiles.

On the other hand, the Froude numbers can provide valuable insights into the nature of the flow, helping to identify potential areas of concern, such as supercritical conditions. In this context, Froude values at zones I and II, generated by Group A, are reported to be much higher than those obtained by Group B, with the exception of the smallest opening height (Fig 11A). The higher Froude numbers in zones I and II for Group A suggest that the flow in these zones is more intense and potentially supercritical, particularly for smaller opening heights. Furthermore, the largest Froude number in zone III, generated by the opening height of 100 cm, suggests a highly supercritical flow.

The boundary velocity is attributed to the averaged velocity of flow around asperities and strongly depends on the thickness of the boundary layer and the types of irregularities in the flow. The consistency observed in the boundary velocity profiles across different opening configurations, excluding the smallest operation case of 25 cm, suggests that there may be a level of uniformity in the flow characteristics near the boundaries (Fig 11B). Regarding two zones I and II, the highest value of boundary velocity is witnessed with the opening height of 100 cm, while the lowest value is generated by the opening height of 50 cm. Moreover, it is noted that as the opening height increases, the boundary velocity also tends to increase at zone III.

**Fig 12:**
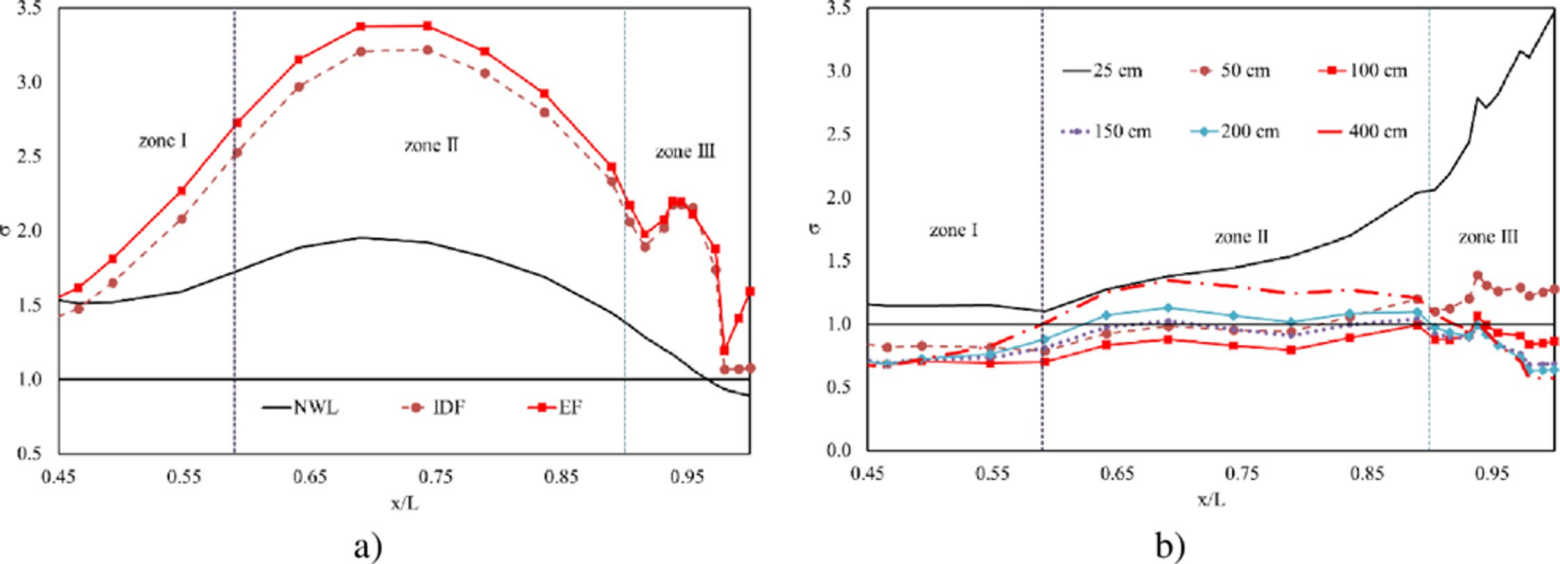
Cavitation index profiles. a) extreme floods; b) operations of controlled gate.

Certainly, cavitation index (*σ*) profiles yielded by three cases of extreme floods are greater than 1.0. That means there is no cavitation damage observed in the studied area (Fig 12A). Meanwhile, among the operations with controlled gates, the smallest opening height does not lead to potential cavitation. However, the opening width of 100 cm causes the highest probability of cavitation, especially at slope segment and curved areas. In contrast, the largest opening height of 400 cm produces the least cavitation risk at these areas but the most potential danger at the flip bucket. However, the smallest value of *σ* for all cases is 0.68, suggesting that potential cavitation may be insignificant (Fig 12B).

**Fig 13:**
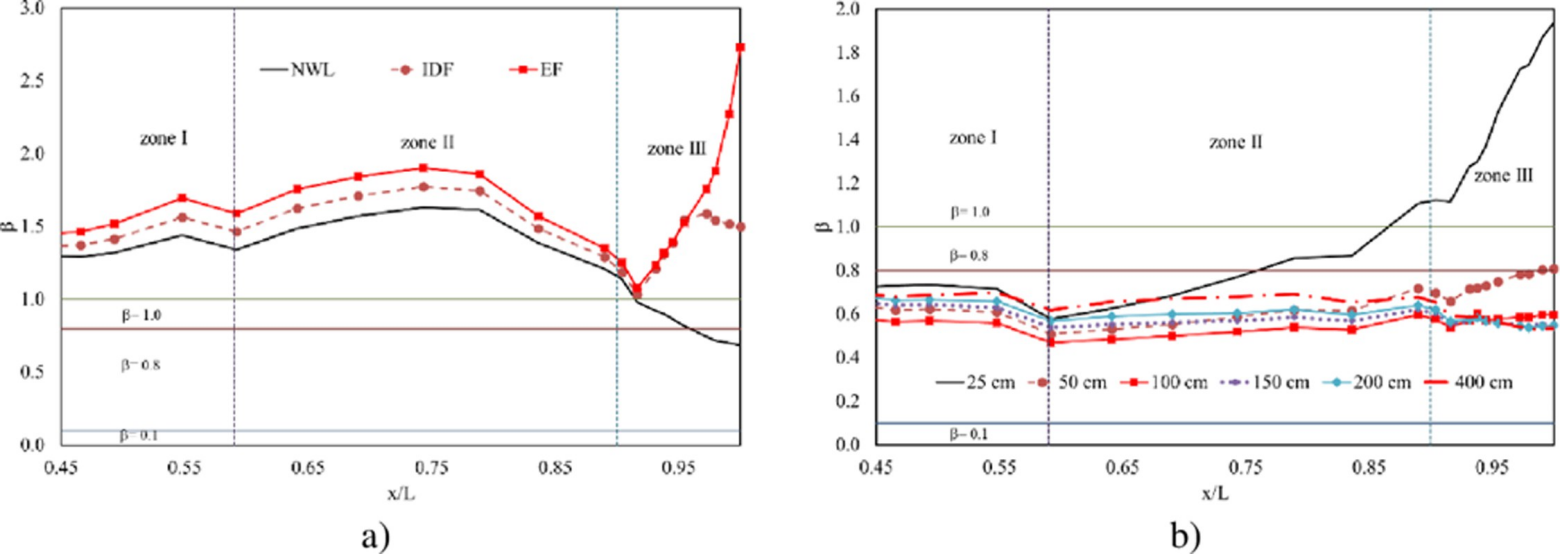
Gasification index profiles. a) extreme floods; b) operations of controlled gate.

Regarding the impact of irregularities, three profiles of gasification index (*β*) generated by a group of extreme floods are much greater than 1.0, except the tail of profile obtained by NWL (Fig 13A). This observation implies that gasification, or the process of forming gas bubbles, does not occur around asperities in these extreme flood scenarios. In contrast, the gasification index (*β*) value profiles are reported to be in the range of 0.55 to 0.8 for a group of opening widths, except for the case of 25 cm. Which means voids or bubbles develop moderately in the studied area (Fig 13B). In addition, the smaller *β* indicates conditions that are more conducive to cavitation, possibly due to the rapid flow of water-mixed sediment. The opening height of 100 cm yields the smallest value of *β* in zones I and II, while the opening height of 400 cm causes the largest value of *β* in these zones. The greater operation height, however, the more potential erosion generates in zone III. This suggests that smaller opening heights cause increase the risk of cavitation in zones I and II and decrease the potential erosion in zone III.

On the other hand, when using the cavitation index *σ*, two opening widths 400 cm and 50 cm may be not associated with potential cavitation risk in zone II and zone III, respectively (Fig 12B). However, the gasification index *β* in these cases is reported to be smaller than 1.0 (Fig 13B). The discrepancy between the *σ* index and the *β* index highlights the importance of considering irregularities in the assessment of cavitation risk. The smaller *β* values suggest that gasification is occurring around asperities, increasing the likelihood of cavitation damage despite the *σ* index suggesting otherwise.

### 3.3. Proposal for the Chay 5 spillway surface rehabilitation

To enhance life service of the aging structure, it is truly necessary to perform the rehabilitation. Based on not only the assessment of cavitation risk as abovementioned, but also the professional observation during the field inspection, the most severe area of the Chay 5 spillway surface that needs to be repaired promptly is the Zone II and III at the curved area and flip bucket. Nevertheless, the rest area of the spillway surface including the Zone I has also to be improved to yield a finish surface as a preventive measure. To do so, the proposed approach might be using a protective layer of specialized mortars applying directly to the existing concrete surface of the spillway [44–50]. Besides, the rehabilitation of the surface would be done in according to the recommendations prescribed in TCVN 9343:2012 [48].

To have the appropriate mortars for rehabilitating, the constituent materials used in this study are cement OPC40, fly ash, silica fume, sand, water and chemical admixtures. Two types of admixture with VMAT brand are used, such as Vmat PC01 and Vmat EXP01. The first one is superplasticizer and the second is a chemical component, which prevents the mortar from shrinking during setting and hardening. Some “trial-and-error” have to be involved to adjust the dosage of water and superplasticizer content. The final mix proportion of the concerned specilized mortars (M30 and M50) is presented in Table 5. Mechanical properties of M30 and M50 are also provided in Table 6. It can be observed that apart from high abrasion resistance, the mortar also possesses high early-age strength, which is suitable for the rehabilitation process that requires a short-time period of execution [48].

**Table 5:** Mix proportion of M30 and M50.

Concrete grade	OPC40 (kg)	Fly ash (kg)	Silica fume (kg)	Sand (kg)	Vmat PC01 (litter)	Vmat EXP01 (kg)	Water (litter)
M30	300	250	-	1450	5	13	175
M50	700	100	100	1100	39	18	350

**Table 6:** Mechanical properties of M30 and M50.

Mortar	Compression strength, MPa			Flexural strength, MPa			Abrasion resistance, g/cm^2^		
	1 day	7 days	28 days	1 day	7 days	28 days	1 day	7 days	28 days
M30	16.5	26.7	34.4	2.51	3.95	4.88	0.38	0.36	0.33
M50	39.8	44.1	56.5	6.31	7.08	9.55	0.22	0.2	0.17

#### 3.3.1. Rehabilitation at the curved area and flip bucket

The Zone II and III at the curved area and flip bucket suffering the most serious damage due to cavitation and abrasion phenomena, caused by the release of high-speed flow of water mixed with sediment over an extended period, require targeted and effective rehabilitation measures. So it is suggested by the expert to use the specialized rehabilitating mortar with high abrasion resistance, anti-shrinkage and the designed strength class of 50MPa or M50 (Table 5) [45, 46].

**Fig 14:**
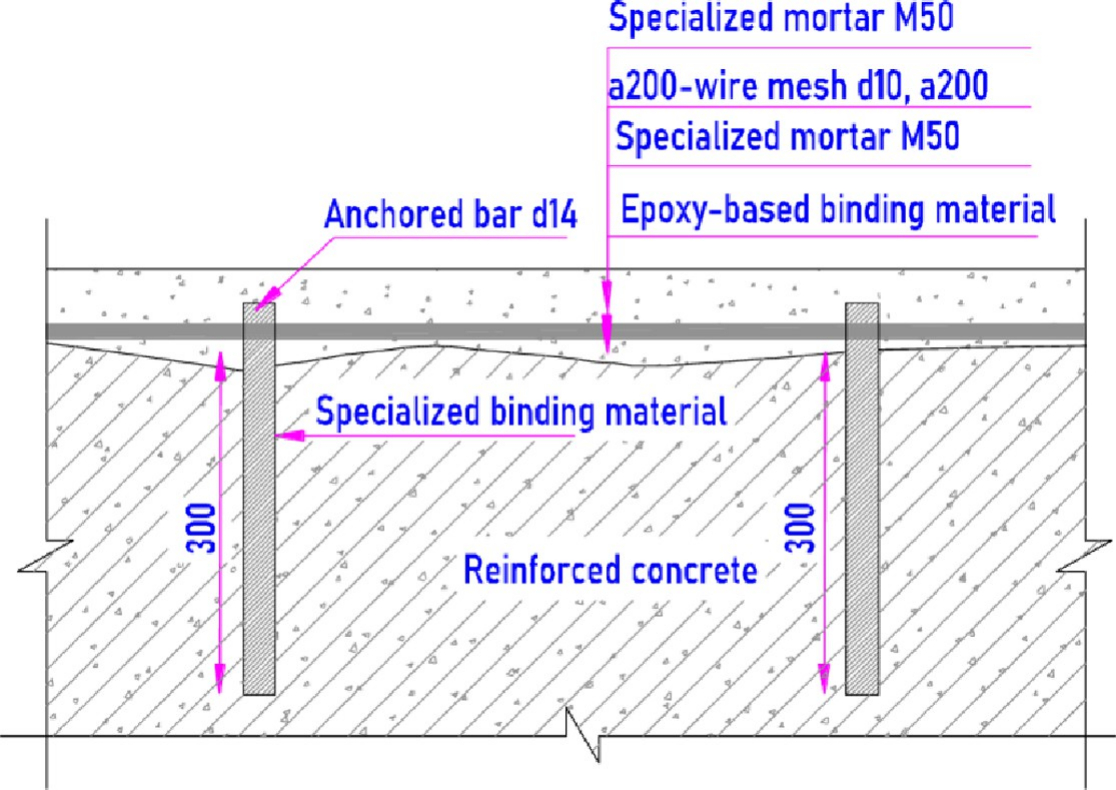
Sketch rehabilitation at zones III.

To conduct the rehabilitation at the curved area and flip bucket, firstly it is necessary to remove weak concrete and create a sound substrate. Moreover, the reinforcement rust of the spillway surface is removed totally, and the bar is aligned and rectified in accordance with the design drawing sketch. The preparation process results in making the pit surrounding the damage location on the surface. After that, a series of holes of diameter 20 mm and depth 300 mm are made inside the pit by a hammer drill. An anchored bar diameter 14 mm is erected inside the hole by using the special binding material. Anchored bars are arranged with a distance of 50 cm one to another on the surface. These anchored bars are interconnected by a wire mesh of steel bar diameter 10 mm and with a grid of 20 by 20. The sectional sketch of the spillway surface after rehabilitation can be observed in Fig 14.

Once the reinforcement is set, M50 is prepared by using a small concrete mixer and then the fresh M50 is placed to fill the pit fully. After M50 placement, the curing process is carried out by covering a plastic onto the surface to avoid the evaporation. As the mortar is hardened properly as designed, the repairing process is finished. Since M50 is workable at the fresh state, it results in a smooth surface after hardening. Without surface roughness and high abrasion resistance of the M50 will diminish the cavitation risk significantly.

#### 3.3.2. The rest area of the spillway surface

**Fig 15:**
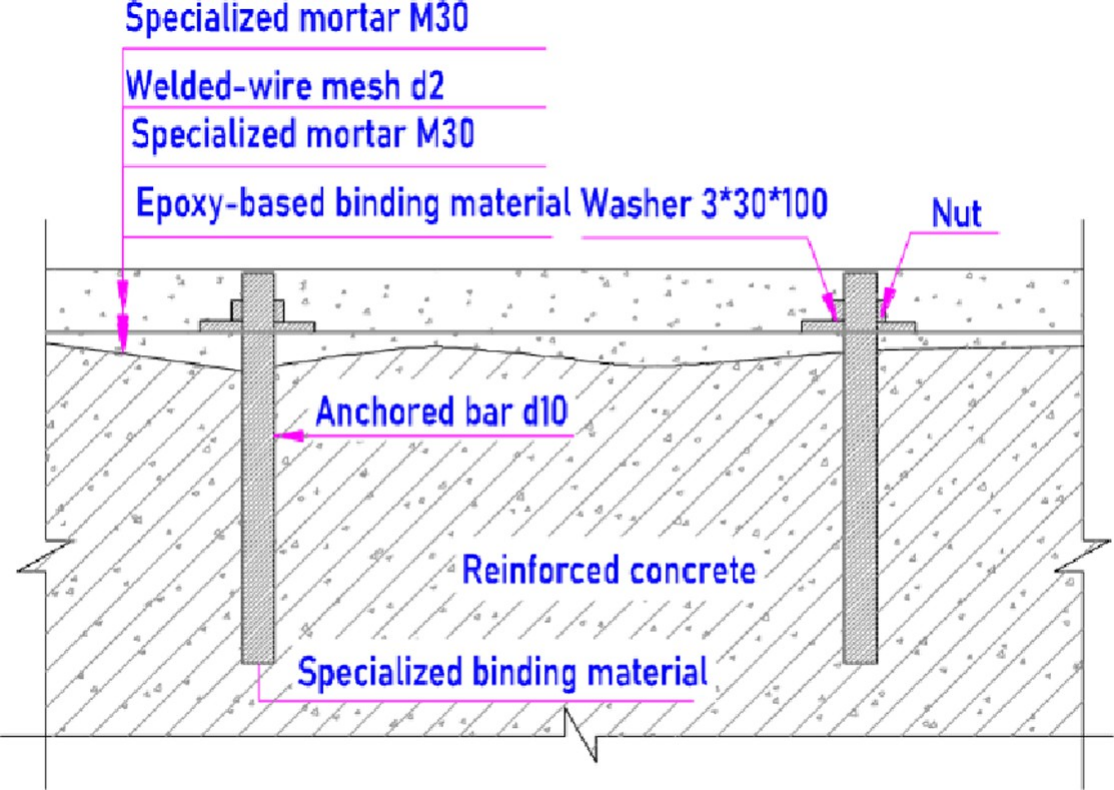
Sketch rehabilitation at the rest of spillway.

Apart from the rehabilitation of the severe damage location at the curved area and flip lip as abovementioned, the rest area of the spillway surface including the Zone I also needs a rehabilitation. To do that, a rehabiting mortar with high abrasion resistance, anti-shrinkage and the designed strength class of 30MPa or M30 (Table 5) is placed onto the surface with a layer thickness of 30 mm, as well as reinforcement is established inside. The reinforcement set is similar to that mentioned above with anchored bars diameter 10 mm and they are embeded into the existing concrete with a depth of 150 mm, as it can be seen in the sectional sketch, which is illustrated in Fig 15.

Anchored bars are arranged with a distance of 50 cm one to another on the surface. The anchored bars are interconnected by a wire mesh of steel bar diameter 2 mm and with a grid of 50 by 50. The bar is fixed to the mesh by using a nut and washer, which has a thickness of 3 mm and 30 mm width by 100 length. Once the Chay 5 spillway surface rehabilitation is done, it promises to be sustained more firmly from the water impact and consequently the service life would be improved crucially.

## 4. Conclusions

The optimal navigation strategy of aging spillway released water mixed sediment under various operational conditions involves a holistic approach that considers hydraulic performance, safety, and efficiency. In this study, the strategy should be suggested: i) Hydraulic modeling and simulation; ii) Risk assessment and optimal gate operation strategies; iii) Maintenance and rehabilitation.

- Hydraulic modeling and simulation by using of 3D numerical models provides a comprehensive and versatile approach in hydraulic engineering. Mesh convergence analysis and validation is necessary step to select the suitable mesh size and obtain accurate numerical results.
- Cavitation risk assessment is used to identify potential failure modes and vulnerabilities associated with spillway operation. While the cavitation index (*σ*) may be suitable for assessing cavitation risk in well-designed hydraulic structures, the gasification index (*β*) becomes more crucial when evaluating cavitation damage risk in aging structures with inevitable irregularities. Considering group of opening height scenarios of Chay 5 spillway, the use of the gasification index (*β*) to analyze cavitation risk on an aging spillway is deemed more suitable than cavitation index (*σ*). Slope and concave segments exhibit that an opening height of 100 cm poses the highest cavitation risk, while an opening height of 400 cm presents the most susceptibility at flip bucket. So, manage the opening height of 100 cm of radial gate during a short-term operation when release water with sediment before a flood event led to reduce cavitation risk at slope and concave segments. However, the navigation of greater opening heights causing erosion damage at flip bucket is inevitable, this area should be inspected and considered for the rehabilitation annually.
- The proposed approach to rehabilitating the entire spillway surface is carried out by using a protective layer of anti-shrinkage high abrasion resistance mortar applying directly to the existing concrete surface of the spillway. The resultant surface after rehabilitation is smooth and even finish. This would promise to reduce the cavitation risk and enhance service life of the structure accordingly.

In summary, both visualization the realistic spillway surface and numerical simulation high-speed flow should be regularly implemented, which contributes to predict accurately the development of cavitation risk. Besides, numerical prediction of the potential cavitation damage area on spillway surface under various navigation controlled gate scenarios can help build the optimal navigation strategy as well as propose sufficient maintenance and rehabilitation methods. This comprehensive risk assessment can be applied to aging conveyance structures, to elongate their service life.

This study still exhibits certain limitations that warrant further investigation: i) The presented numerical models cannot simulate long-term abrasion phenomena due to the limitation of computational resources. ii) The presented model is unable to simulate water mixed sediment flow due to insufficient information about sediment properties, potentially leading to less precise hydraulic results. However, to address the issues of sediment flow-induced abrasion and erosion on concrete surfaces, the construction of a bottom outlet for sediment flushing is indeed a critical measure.

## Supporting information

S1 File Chay 5 spillway design. (PDF)

## Abbreviations

ρ: Density of fluid (kg/m^3^)
v: Velocity vector (m/s)
t: Time (s)
p: Pressure (Pa)
p_o_: Absolute pressure at concrete surface (Pa)
p_v_: Vapor pressure (Pa)
g: Gravitational acceleration vector (m/s^2^)
μ: Dynamic viscosity of the fluid (N.s/m)
h: Flow depth (m)
h_v_: Vapor pressure head (m)
▽: Divergence, gradient operator (-)
b: Width of a vent of spillway (m)
K: Coefficient of bubble generation (-)
K_c_: Critical value of bubble generation (-)
β: Gasification index (-)
σ: Cavitation index (-)
V_b_: Velocity at boundary of structure or at the bottom of surface (m/s)
δ: Boundary thickness (m)
△: Height of roughness (m)
V: Depth–averaged velocity (m/s)
ξ_1_: Parameter which depend on the ratio *δ/Δ* (-)
ξ_2_: Parameter which depend on the ratio (*Z*_*m*_+*Δ*) */Δ* (-)
ϕ_v_: Parameter of relation between depth averaged velocity and maximum velocity (-)
B: Width of the rectangular cross-section (m)
L_m_: Width of an asperity (m)
Z_m_: Height an asperity (m)
H: Head on spillway (m)
V_0_: Approach velocity (m/s)
H_0_: Total energy head above crest (m)
P: Height of weir (m)
ε: Side contraction parameter (-)
m: Discharge coefficient (-)
E: Relative error (%)
Q_model_: Numerical volume flow rate (m^3^/s)
Q_theory_: Theoretical discharge (m^3^/s)
OPC40: Ordinary Portland Cement with strength class 40MPa
M30: Mortar strength class 30MPa
M50: Mortar strength class 50MPa
