# Supplementary material for: Evaluation navigation controlled gate of aging spillway on cavitation damage
Source: PLoS One. 2024 Oct 9;19(10):e0311247. doi: 10.1371/journal.pone.0311247 (PMC11463738; doi:10.1371/journal.pone.0311247)
Supplement: S1 File — (PDF) [file pone.0311247.s001.pdf]

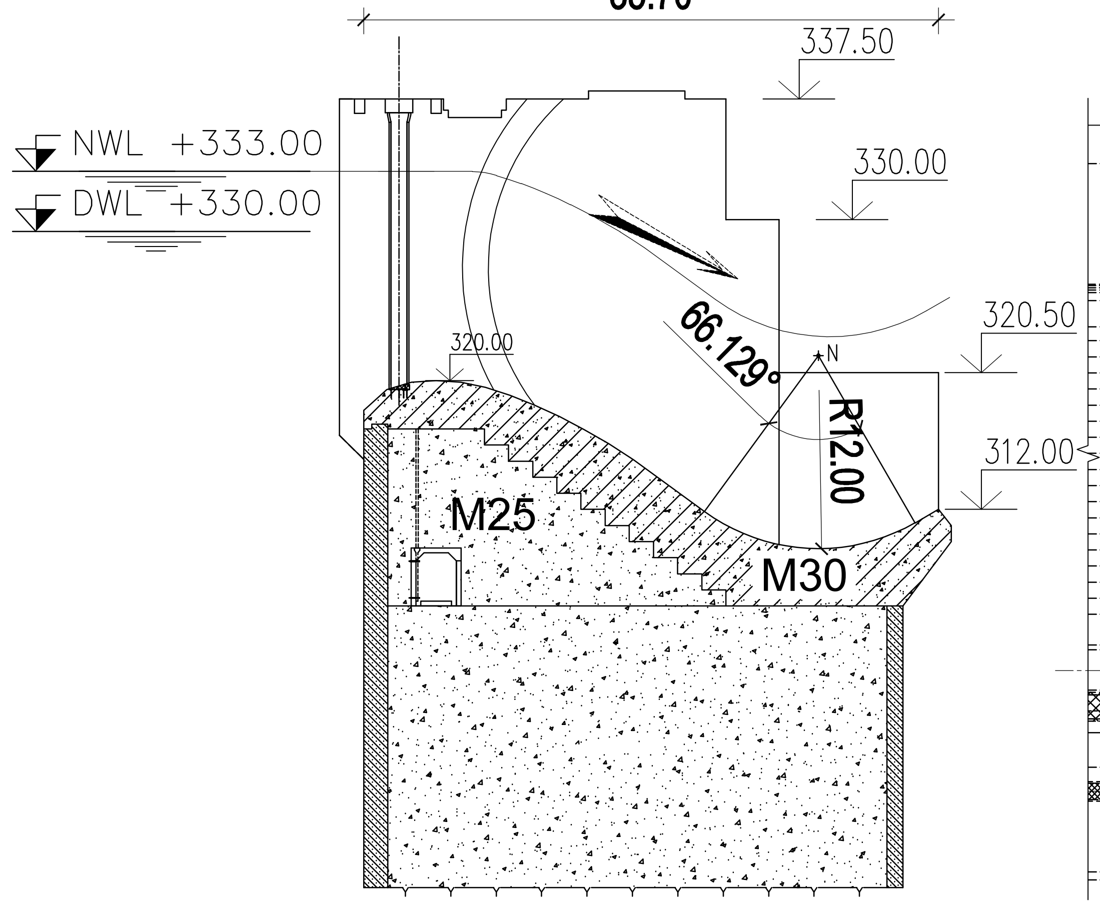

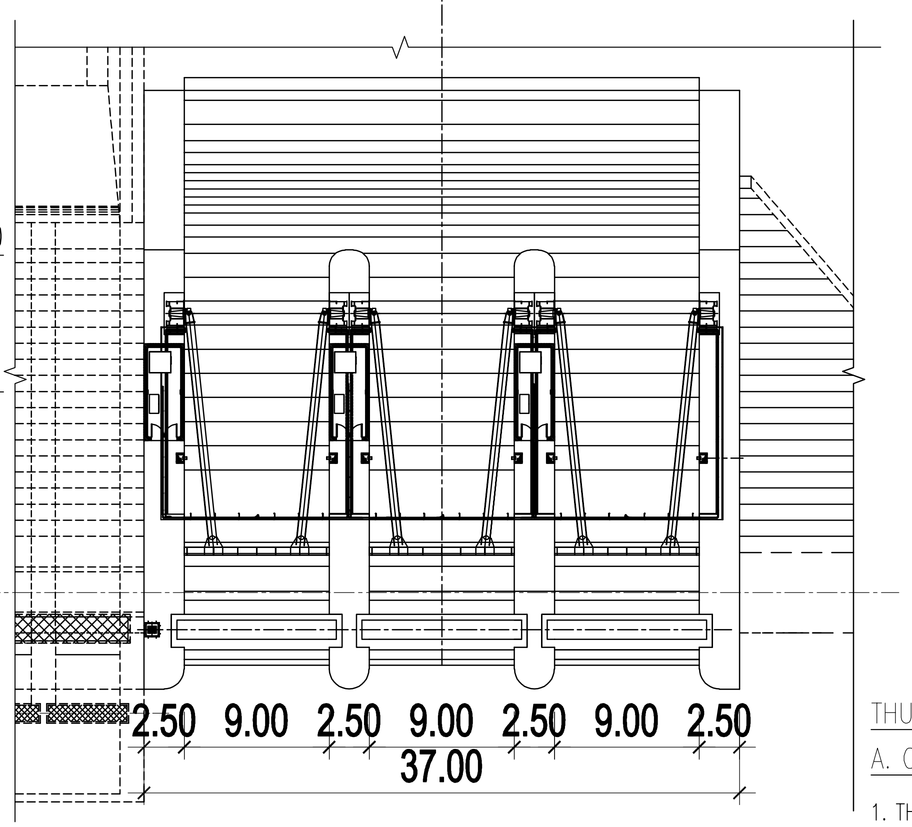

BẢNG TOẠ ĐỘ CÁC ĐIỂM

ĐIỂM	TOẠ ĐỘ	
	X(M)	Y(M)
1	0.00	1.85
2	1.44	0.55
3	2.97	0.11
4	4.50	0.00
5	6.03	0.09
6	7.57	0.41
7	9.10	0.92
8	10.63	1.53
9	12.16	2.24
10	13.69	3.03
11	15.22	3.92
12	16.75	4.91
13	21.17	8.14
14	28.24	10.45
15	34.24	8.84
16	35.70	8.00
N	28.24	1.55
$1-2^{0}$	3.28	4.05
$2-3^{0}$	4.25	7.38
$3-4^{0}$	4.56	11.38
$4-5^{0}$	4.64	10.55
$5-6^{0}$	3.88	14.15
$6-7^{0}$	0.06	25.73
$7-8^{0}$	–2.37	31.79
$8-9^{0}$	–3.34	33.90
$9-10^{0}$	–4.58	34.09
$10-11^{0}$	–3.30	36.29

ĐƯỜNG CONG MẶT TRÀN KHÔNG CHÂN KHÔNG – OFITXEROV

TỶ LỆ: 1/500

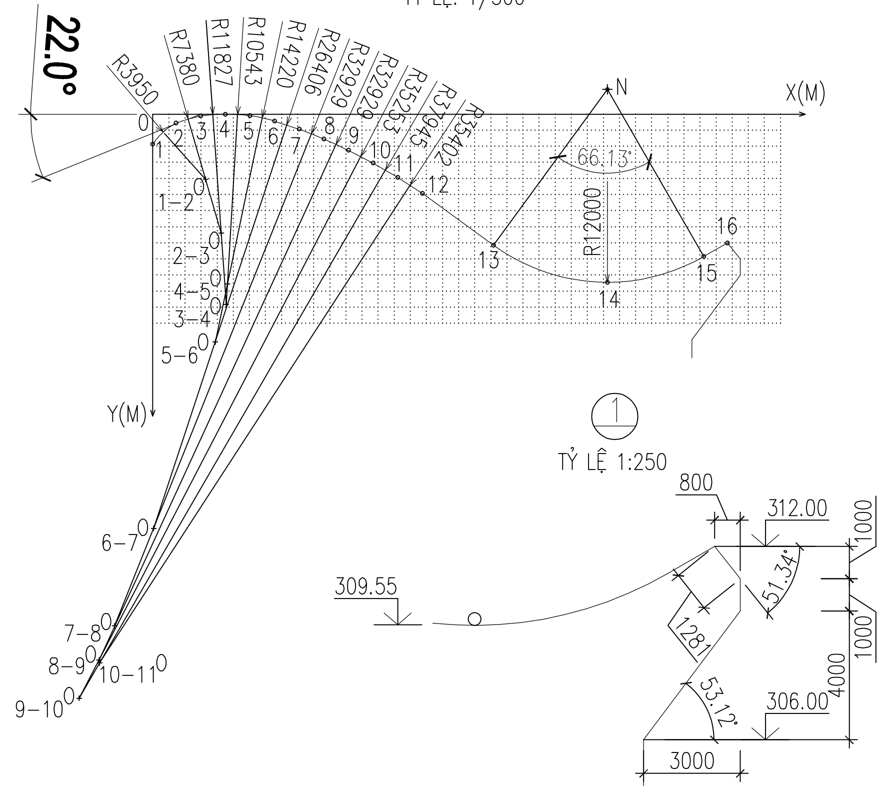

GHI CHÚ:

1. KÍCH THƯỚC TRONG BẢN VẼ GHI BẰNG MM, CAO ĐỘ GHI BẰNG M.
2. BẢN VẼ NÀY XEM CÙNG CÁC BẢN VẼ: SC5–ĐT–06–01–:–20
3. CHỈ DẪN CHUNG, TỔNG HỢP KHỐI LƯỢNG XEM BV: SC5–ĐT–06–02

THUYẾT MINH THIẾT KẾ:

A. CĂN CỨ ĐỂ LẬP BẢN VẼ:

1. THIẾT KẾ NÀY LẬP TRÊN CƠ SỞ THIẾT KẾ KĨ THUẬT – CÁC BẢN VẼ PHẦN XÂY DỰNG DO CÔNG TY CỔ PHẦN TƯ VẤN XÂY DỰNG VÀ THƯƠNG MẠI TÂY NGUYÊN ĐÀ LẬP (05/2010).
2. CÁC TÀI LIỆU ĐỊA HÌNH, ĐỊA CHẤT DO CÔNG TY CỔ PHẦN TƯ VẤN CÔNG NGHỆ VÀ XUẤT NHẬP KHẨU LINKERVN LẬP THÁNG 08/2009
3. CÁC TIÊU CHUẨN, QUI PHẠM ... HIỆN HÀNH.

B. THUYẾT MINH THIẾT KẾ XÂY DỰNG:

1. BỘ BẢN VẼ NÀY THIẾT KẾ BÊ TÔNG MẶT TRÀN, MŨI PHÓNG.
2. CÔNG TÁC THI CÔNG VÀ NGHIỆM THU PHẢI TUÂN THEO CÁC ĐIỀU KIỆN KỸ THUẬT THI CÔNG
3. CÔNG TÁC CHUẨN BỊ NỀN KHỐI ĐỔ:
   - LOẠI BỎ TẤT CẢ ĐẤT ĐÁ, RÁC BẨN V.V... RA KHỎI NỀN KHỐI ĐỔ.
   - VỆ SINH RỬA NƯỚC, THỔI KHÍ NÉN NỀN, BỀ MẶT KHỐI ĐỔ ĐỂ ĐẢM BẢO BỀ MẶT SẠCH VÀ KHÔNG CÒN ĐỌNG NƯỚC.
4. BÊTÔNG MẶT TRÀN SỬ DỤNG BÊ TÔNG M300, B10, R135, DMAX=20MM, ĐỘ SỤT OK = 4–6.
   BÊTÔNG TƯỜNG TRỤ BIÊN, TRỤ PIN SỬ DỤNG BÊ TÔNG M300, B10, R135, DMAX=40MM, ĐỘ SỤT OK = 4–6.
5. BỀ MẶT BÊ TÔNG MẶT TRÀN ĐƯỢC HOÀN THIỆN Ở CẤP ĐẶC BIỆT F3.
   BỀ MẶT BÊ TÔNG TƯỜNG TRỤ BIÊN, TRỤ PIN ĐƯỢC HOÀN THIỆN Ở CẤP CAO F2.
6. THI CÔNG CỐT THÉP TẠI CHỖ HOẶC TRONG XƯỞNG PHẢI TUÂN THEO TCVN – 4453 – 1995.
7. CHIỀU DÀY LỚP BẢO VỆ CỐT THÉP LÀ 50 MM TÍNH TỪ TÂM CỐT THÉP NGOÀI CÙNG ĐẾN BIÊN BÊ TÔNG.
8. KHÔNG ĐƯỢC BỐ TRÍ ĐOẠN NỐI BUỘC THÉP TẠI NHỮNG ĐOẠN UỐN CONG HAY VỊ TRÍ CHỊU LỰC LỚN, TRONG MỘT MẶT CẮT NGANG CỦA TIẾT DIỆN KẾT CẤU KHÔNG ĐƯỢC NỐI THÉP QUÁ 50%.
9. KHI THI CÔNG CẦN ĐẶT TẤT CẢ CÁC CỐT THÉP CHỜ ĐỂ ĐẢM BẢO LIÊN KẾT VỚI CỐT THÉP CÁC KHỐI XUNG QUANH.

THUỶ ĐIỆN SÔNG CHẢY 5-TỈNH HÀ GIANG

HẠNG MỤC: ĐẬP TRÀN
MỤC: BÊ TÔNG MẶT TRÀN - MŨI PHÓNG
MẶT BẰNG VỊ TRÍ, MẶT CẮT A-A

01/151 **SC5-ĐT-06-01**
